# Lunatic Asylums in the Bombay Presidency

**Published:** 1902-11-08

**Authors:** 


### LUNATIC ASYLUMS IN THE BOMBAY PRESIDENCY.

Throughout the whole of the six asylums of the Bombay Presidency a considerable increase in the amount of sickness was noticeable during the year 1900. Apparently overcrowding has not been to blame for this unfortunate state of affairs. Except on the female side of the Colába Asylum, in every case the accommodation provided has been amply sufficient for the number of patients desiring admission. With regard to the Colába Asylum it is quite true that the death-rate was exceptionally heavy, but it was particularly so amongst the men. Strangely enough, too, comparing the statistics of the five previous years, it is noticeable that the death-rate was distinctly less when the overcrowding was greater. But the fact remains that overcrowding on the female side of this particular hospital has existed, more or less, for the past ten years, and it is time that the authorities took steps to remedy such a state of affairs. Otherwise, as far as structural defects and general organisation and equipment go, the results of the management of these institutions has been satisfactory, and creditable to the medical officers and their subordinates. Poona is badly in want of a new asylum, which is already under discussion. The arrangements in the present institution are far from perfect. The buildings remain unsuitable and ill-arranged; and as in some other asylums there is no separation possible of criminals, juveniles or patients with various forms or phases of insanity, and the recreation yards are small and confined. The sums expended during the year for the amusement of the patients in the asylums of the Bombay Presidency was Rs.1,476, as compared with Rs.1,380 in the previous year. All the inmates capable of doing useful occupations are practically employed; but there is an absence of facilities for occupation, and also a great lack of intelligent supervision. Both these facts are to be deplored, and if matters were bettered the benefit would probably be almost at once apparent in the improvement of the mental health of the inmates. At present the percentage of cures to the daily average strength is only—males 10·6, and females 5·1. There were three escapes during the year, all non-criminals, and all inmates of the asylum at Hyderabad. One was recaptured, two are still at large. The walls in this asylum are very low, and it would have been thought that noting how easily the inmates can make their escape, something would be done to heighten them at once; but when the last report was issued the authorities had got no farther than suggestions. The cost of all the asylums was considerably higher than usual owing to the dearness of provisions in 1900, the expense for the year for diet being Rs.80.12.1 per head instead of Rs.76.0.4. With an average daily strength of 779 this increase naturally accrues to a heavy total.

